# An Electronic Health Record–Based Strategy to Systematically Assess Medication Use Among Primary Care Patients With Multidrug Regimens: Feasibility Study

**DOI:** 10.2196/resprot.7986

**Published:** 2017-08-10

**Authors:** Stacy Cooper Bailey, Christine U Oramasionwu, Alexandra C Infanzon, Emily R Pfaff, Izabela E Annis, Daniel S Reuland

**Affiliations:** ^1^ Division of Pharmaceutical Outcomes and Policy Eshelman School of Pharmacy University of North Carolina at Chapel Hill Chapel Hill, NC United States; ^2^ North Carolina Translational and Clinical Sciences Institute School of Medicine University of North Carolina at Chapel Hill Chapel Hill, NC United States; ^3^ Division of General Medicine and Clinical Epidemiology Department of Medicine University of North Carolina at Chapel Hill Chapel Hill, NC United States

**Keywords:** patient portal, medication adherence, health literacy

## Abstract

**Background:**

Medication nonadherence and misuse are public health and patient safety concerns. With the increased adoption of electronic health records (EHRs), greater opportunities exist to communicate directly with, and collect data from, patients through secure portals linked to EHRs.

**Objective:**

The study objectives were to develop and pilot test a method of monitoring patient medication use in outpatient settings and determine the feasibility and acceptability of this approach.

**Methods:**

Adult primary care patients on multidrug regimens were recruited from an academic internal medicine clinic by a trained research assistant. After completing a baseline, in-person interview, patients were sent a link to a questionnaire about medication use via the patient portal. One week later, the RA contacted patients to complete a follow-up telephone interview assessing patient satisfaction and experience with the questionnaire. Patient EHRs were also reviewed to determine the questionnaire completion rate.

**Results:**

Of 100 patients enrolled, 89 completed the follow-up interview and 82 completed the portal questionnaire. The mean age of the sample was 61.8 (range 31-88) years. Approximately half (54/100, 54%) of the sample was male, two-thirds were white (67/100, 67%) and 26% (26/100) African-American. A total of 44% reported an annual household income of <$50,000 per year, and 17% (17/100) reported a high school or less level of education. No significant differences were found in questionnaire completion rates by sociodemographic characteristics or prior portal use. Most (68/73, 93%) found the questionnaire easy to access, easy to complete (72/73, 99%), and valuable (73/89, 82%). Time constraints and log-in difficulties were the main reasons for noncompletion.

**Conclusions:**

The portal questionnaire was well received by a socioeconomically diverse group of patients with high completion rates achieved. Routine use of a portal-based questionnaire could provide a valuable signal to providers and care teams about patient medication use and identify patients needing additional support.

## Introduction

While medication nonadherence has long been recognized as a public health and patient safety concern, it is likely to become increasingly important with the growing prevalence of chronic disease and the aging of the US population [1-3]. Estimates indicate the number of people taking prescription drugs is increasing over time, with the percentage of the US population who take 5 or more medications nearly doubling from 8% in 1999-2000 to 15% in 2011-2012 [4,5]. Such complex, multidrug regimens usually require greater patient self-management skills and clinical oversight. However, providers often lack the time and resources needed to identify and assist patients who exhibit poor medication adherence or misunderstand instructions for use.

With the increased implementation and use of electronic health records (EHRs), greater opportunities exist to engage patients in care and collect patient data on health behaviors outside the clinic setting [6]. Yet while the potential value of using EHRs for patient engagement and monitoring has been well recognized, the actual acceptability and practicality of EHR-based strategies have not been as well explored [7,8]. In this feasibility study, we sought to leverage a commonly used EHR platform to develop and pilot test a strategy to monitor patient medication use in outpatient settings and determine the feasibility and acceptability of this approach. Findings from this study can inform future implementation and use of similar EHR-based tools.

## Methods

### Envisioning the Electronic Health Record–Based Strategy

The intent of this study was to develop and pilot test a low-cost, sustainable method of systematically collecting data from patients on their prescription medication use at home. The belief was that such data could help identify patients who were struggling with medication adherence or had other medication concerns that could reasonably impact their health care and health outcomes. Given limited clinic resources, a portal-based questionnaire, which could be automatically delivered to any patient or group of patients on a routine basis and for virtually no cost, was selected as the most appropriate means of collecting patient data on medication use outside the clinic visit. The strategy was envisioned to include 3 steps: (1) patients receive and complete an online questionnaire via the patient portal, (2) questionnaire results are recorded in the EHR for review by the clinical care team, and (3) based upon results, the clinical care team provides additional counseling or resources to patients on appropriate medication use according to a clinic protocol. The study reported herein is focused on the development and initial pilot-testing of the portal questionnaire itself (steps 1 and 2); additional studies are planned to evaluate the strategy as a whole.

### Building the Electronic Health Record Strategy

To create the portal questionnaire, a survey consisting of 12 items from the Measure of Drug Self-Management (MeDS) was built using Epic EHR (Epic Systems Corp) [9]. The MeDS, which deconstructs the tasks associated with taking medications focusing on the patient knowledge, skills, and behaviors needed to correctly take multiple drugs, was chosen because it was originally designed for use in an EHR environment. Items and response options were programmed individually and then grouped together into a survey. The questionnaire was designed to be delivered to a patient via My Chart, the health care system’s patient portal powered by the Epic EHR system, along with an accompanying new message notification sent to the patient’s on-file email address. A link was included in the message to direct patients to the questionnaire.

After completion, individual item responses were tallied and an overall score was calculated and filed in the patient’s chart for viewing by the clinical care team. The intention was for questionnaire results to help inform patient-provider discussions on medication use during future clinical appointments and/or to trigger immediate outreach to the patient should a serious medication concern be identified. The exact response would be determined by the nature of the questionnaire results and clinical care protocols. The provider’s view within the EHR was also custom built to allow questionnaire results to be graphed longitudinally over time along with other relevant patient data. This presentation was chosen as it would enable providers to monitor trends in medication use and determine the effects, if any, of clinical interventions to improve adherence.

### Pilot-Testing the Electronic Health Record–Based Strategy

#### Overview

Following its development, this EHR-based strategy was pilot-tested among patients receiving care at an academic general internal medicine clinic. Convenience sampling was used to recruit patients. Specifically, a research assistant reviewed the list of daily appointments at the study clinic and approached patients who were potentially eligible (by age, medications prescribed, and patient portal access) to introduce the study and determine patient interest and eligibility. Patients were considered eligible if they were age 18 years or older; were English-speaking; had primary responsibility for managing their own medications; were prescribed at least 3 medications including 2 specific medications for diabetes and high cholesterol (ie, metformin, atorvastatin); had access to high-speed Internet at home or had a smartphone; were a clinic patient with an activated patient portal account; and had no severe cognitive, visual, or hearing impairment that would preclude informed consent or study participation.

After verifying eligibility, the RA engaged the patient in the informed consent process and conducted a structured, in-person interview. The RA then manually assigned the questionnaire to the participant within the portal. A follow-up telephone interview was conducted 1 week later by the same RA. Participants were compensated $20. An institutional review board approved study procedures.

#### Outcomes

Process outcomes for this study included whether the questionnaire was delivered to participants via the portal, whether participants completed the questionnaire, and whether the questionnaire results were recorded and displayed correctly within the EHR. The RA recorded outcomes by abstracting data from the EHR. Additionally, participants were asked to self-report receipt and completion of the questionnaire during the follow-up interview. The interview also assessed patient experiences with and perceptions of the electronic tool and strategy.

#### Sociodemographic Variables and Covariates

The baseline battery also included questions measuring key patient health and sociodemographic characteristics as well as prior portal and Internet use. Health literacy was measured using the Rapid Estimate of Adult Literacy in Medicine (REALM), a word pronunciation test that is commonly used to assess patient literacy skills [10].

### Statistical Analyses

Descriptive statistics were calculated for patient sociodemographic variables and study outcomes. To assess whether there were any systematic, statistically significant differences between patients who completed the follow-up interview and those who did not, we used Pearson chi-square test or Fisher exact test for categorical variables and Student *t* test for age. The same tests were used to compare the differences between the patients who completed the portal questionnaire and those who did not. Specifically, we examined if completion varied by age, sex, race/ethnicity, education, income, literacy skills, and average use of the patient portal. We used α=.05 to determine statistical significance. All analyses were performed using SAS version 9.3 (SAS Institute Inc).

## Results

### Participant Characteristics

Recruitment took place from March 2016 to August 2016. A total of 171 patients were approached; 39 patients declined, 31 patients were ineligible, and 1 patient consented but did not initiate the interview. A total of 100 patients completed the baseline interview.

Table 1 describes the characteristics of the study sample (N=100). The mean age of this study sample was 61.8 (range 31-88) years. Approximately half (54/100, 54%) of the sample was male, two-thirds were white (67/100, 67%), and 17% (17/100) reported a high school or less level of education. Most (92/100, 92%) participants had adequate literacy skills according to the REALM. Few patients (5/100, 5%) reported never having used the patient portal; 39% (39/100) stated that they used the portal once per month or less. Most patients (62/100, 62%) reported having used a computer to access the site, 19% (19/100) reported using a smartphone, 13% (13/100) a tablet, and 21% (21/100) said they used all 3 types of devices (categories not mutually exclusive). We observed no sociodemographic differences between patients who participated in the follow-up interview (n=89) versus those who did not (n=11).

### Process Outcomes

A review of the EHR revealed that the portal questionnaire was successfully generated and delivered to all participants (N=100). Of those, 82 of 100 participants completed and submitted the questionnaire via the portal; all questionnaire scores and item responses were recorded and displayed correctly in patient charts.

A total of 73 participants who completed the questionnaire also completed the follow-up interview. Of these, the majority (65/73, 89%) reported completing the portal questionnaire on a computer, laptop or tablet while the remaining 11% (8/73) patients reported completing the measure via a smartphone. There were no sociodemographic differences between participants who completed the portal questionnaire versus those who did not (Table 2). Similarly, patients who reported never having used the patient portal or using it less than once per month were as likely to complete the portal questionnaire as more frequent patient portal users (*P*=.61).

### Satisfaction with the Questionnaire

Most patients (68/73, 93%) who completed both the questionnaire and the follow-up interview reported that the tool was very easy to access on the portal, and 99% (72/73) of patients reported the tool was very easy to complete. Most patients reported that the questionnaire was an acceptable length, with only 2 patients stating that it was too long. Overall, satisfaction with the questionnaire was high, with the tool receiving an average satisfaction score of 9.3 (range of 5 to 10), with a score of 10 indicating highly satisfied.

### Reasons for Noncompletion

A total of 16 people completed the follow-up interview but did not complete the portal questionnaire. The majority of these participants (10/16) cited time constraints as the reason for not completing the tool. A total of 3 participants reported that they had difficulty logging into the portal, and 2 stated that they did not receive a notification email. The remaining patient did not provide a specific reason for noncompletion.

### Participants’ Perceived Usefulness of the Electronic Health Record Strategy

Of the patients participating in the follow-up interview, 82% (73/89) believed that a medication adherence questionnaire would be valuable to complete prior to clinic visits to help measure and track patient medication self-management skills over time. While 16% (14/89) of patients stated that they did not know if the questionnaire would be valuable, 2% (2/89) of respondents stated that they did not believe such a tool would be useful.

**Table 1:** Characteristics of study sample.

Variable		Participants N=100
Age, years, mean (range)		61.8 (31-88)
**Sex, n**		
	Male	54
	Female	46
**Race/ethnicity, n**		
	African American	26
	White	67
	Other	7
**Educational attainment, n**		
	High school diploma, general education diploma, or less	17
	Some college	26
	College degree or more	57
**Income, n**		
	<$19,999	16
	$20,000 to $49,999	28
	≥$50,000	45
	Don’t know/refused	11
**Literacy skills, n**		
	Inadequate	8
	Adequate	92
**Methods of prior portal access, n^a^**		
	Never accessed	5
	Computer or laptop	62
	Tablet	13
	Smartphone	19
	All above devices	21

^a^Responses are not mutually exclusive.

**Table 2:** Patient characteristics, stratified by completion of portal questionnaire.

Characteristic			Completed N=82		Not completed N=18		*P* value^a^
Age, years, mean (SD)			61.6 (11.8)		62.6 (12.0)		.74
**Sex, n (%)**							.50
	Male	43 (52)		11 (61)			
	Female	39 (48)		7 (39)			
**Race/ethnicity, n (%)**							.09
	White	58 (71)		9 (50)			
	Other	24 (29)		9 (50)			
**Educational attainment, n (%)**							.45
	High school diploma, general education diploma, or less	12 (15)		5 (28)			
	Some college	22 (27)		4 (22)			
	College degree or more	48 (59)		9 (50)			
**Income, n (%)**							.19
	<$50,000	34 (42)		10 (56)			
	≥$50,000	40 (49)		5 (28)			
	Don’t know/refused	8 (10)		3 (17)			
**Average patient portal use, n (%)**							.61
	Once per month or less	31 (38)		8 (44)			
	2-3 times per month	33 (40)		5 (28)			
	At least once weekly	18 (22)		5 (28)			

^a^Differences between groups were tested using Student *t* test for continuous variables and Pearson chi-square test or Fisher exact test for categorical variables.

## Discussion

### Principal Findings

Results from this feasibility study indicate that a patient portal-based questionnaire could be used among primary care patients to routinely and systematically assess medication self-management skills and identify medication-related concerns. Most patients found a portal-based questionnaire to be easy to use, and high completion rates were achieved among a sociodemographically diverse set of patients. Of note, only 18 of the 100 patients who were sent the questionnaire did not complete it. Analyses suggest that there were no systematic differences between those who completed the portal questionnaire and those who did not; the most commonly cited reason for not completing the online assessment was lack of time, not difficulty with the tool itself. The majority of patients reported believing that the EHR-based strategy could be beneficial in terms of keeping their providers informed of their outpatient medication use.

While these findings are promising, it is necessary to note that portal-based tools are only likely to be beneficial to those who can use them, namely, those who have access to the Internet and have the cognitive and computer skills necessary to complete an online assessment. Individuals lacking Internet access have historically been more likely to be older, low-income, members of racial/ethnic minority groups, and to have limited literacy skills, raising concerns that implementing EHR-based interventions may further exacerbate disparities [11,12]. However, recent national data suggests that Internet access is on the rise among many of these groups, particularly when access via smartphones is taken into account [13,14]. An estimated 77% of US adults now report that they own a smartphone, with similar rates of ownership among white, African American, and Hispanic adults [15]. Use of mobile devices may therefore help reduce racial disparities in portal access and increase overall use. This is reflected in this study, where 11% of participants completed the portal-based assessment via their smartphone and 40% reported having used a smartphone (either as the sole method or one of multiple methods) to access the portal in the past.

Beyond increasing accessibility via mobile devices, other advances could also promote patient use of EHR-based tools. Recently deployed technology at this study clinic will now allow for electronic questionnaires to be completed securely by patients outside the portal environment, with the results still populating in patient charts. This is likely to remove some of the noted barriers to completing portal-based assessments, such as difficulties logging into the portal or remembering portal passwords.

### Limitations

There are limitations to the study that should be noted. First, it was a small feasibility study conducted among 100 primary care patients at one university-based clinic. All patients enrolled in the study had Internet access and a portal account, although the account may not have been in active use. Results may not be generalizable to other populations. We were also limited in terms of the analyses that could be conducted given the small sample size and lack of variability in certain outcomes. In particular, we were unable to determine the acceptability of this approach specifically among patients with limited health literacy. Additional studies will need to be conducted to determine if this strategy is suitable for this patient population.

As this was a preliminary feasibility study, we did not assess provider access and use of questionnaire results nor did we examine its effectiveness or long-term impact in actual use. Informal feedback from health care providers at the study clinic indicated that many believed the questionnaire could be beneficial, although they acknowledged that physicians often experience information overload and may have difficulty reviewing scores and responding to identified concerns during time-limited clinic visits. It is also unknown whether the utility of the measure would diminish over time after multiple administrations or if it would be improved if tailored to patients based on their own unique, medication-related challenges. Additional research will be needed to examine the effectiveness of the EHR-based approach and its utility to patients, providers, and care teams.

### Conclusions

Routine use of a portal-based questionnaire could provide a valuable signal to providers and their care teams about patient use of medications and help identify those patients in need of additional counseling or resources. Such an approach could help improve quality of care and promote medication adherence and safety as questionnaire results could alert providers to patient confusion that could lead to nonadherence, potential medication errors or even a preventable adverse drug event. This strategy could also help elucidate if a patient’s inability to achieve therapeutic goals is related to biologic failure of the drug regimen or poor self-care. While more research is needed, preliminary results from this study indicate portal questionnaires can be successfully generated, delivered to, and completed by patients with results stored in charts for subsequent review and monitoring by members of the primary health care team. Additional research will be necessary to fully evaluate the strategy in actual use.

## Abbreviations

EHR: electronic health record
MeDS: Measure of Drug Self-Management
REALM: Rapid Estimate of Adult Literacy in Medicine
